# United States Medical Licensing Examination Step 2 Clinical Knowledge and Research Productivity in Dermatology Residency Applications in the Post-Step 1 Era: Analysis of Match Outcomes From 2020 to 2024

**DOI:** 10.7759/cureus.87553

**Published:** 2025-07-08

**Authors:** Kevin T Nguyen, Ryan Koch, Kritin K Verma, Megan Nguyen, Jonathan Aldrete, Helen Chen, Justin Raman, Daniel P Friedmann, Ethan Matthew, Michelle B Tarbox

**Affiliations:** 1 School of Medicine, Texas Tech University Health Sciences Center, Lubbock, USA; 2 School of Medicine, Texas A&M University, College Station, USA; 3 Dermatology, Texas Tech University Health Sciences Center, Lubbock, USA; 4 Westlake Dermatology Clinical Research Center, Westlake Dermatology & Cosmetic Surgery, Austin, USA

**Keywords:** application, dermatology, research, residency, usmle step 2 ck

## Abstract

In recent years, the evaluation metrics for dermatology residency applications have changed, particularly following the transition of the United States Medical Licensing Examination (USMLE) Step 1 to a pass/fail grading system. This retrospective analysis examines dermatology residency match data from 2020 to 2024, focusing on trends in USMLE Step 2 Clinical Knowledge (CK) scores and research publications. Data extracted from the National Resident Matching Program documents revealed a notable increase in unmatched applicants with Step 2 CK scores above 250 and a decrease in matched applicants with similar scores. Concurrently, research output has risen, with matched applicants reporting over 25 publications increasing from 80 in 2020 to 121 in 2024, and a corresponding increase among unmatched applicants from 10 to 35 over the same period. Despite these improvements in individual metrics, the overall match rates for applicants with high Step 2 CK scores and extensive research portfolios have declined, suggesting that excellence in these areas alone does not guarantee a successful match.

## Introduction

Dermatology is often recognized as one of the most competitive specialties to apply to for residency [1,2]. Over the past few years, the landscape of dermatology residency applications, like many other specialties, has evolved significantly. Traditionally, factors such as the United States Medical Licensing Examination (USMLE) Step 1 scores, clerkship grades, and letters of recommendation were the important metrics used to evaluate candidates [2]. However, recent shifts in both examination scoring systems and the emphasis on scholarly activities have led to a reassessment of what defines a strong applicant [3].

The transition of the USMLE Step 1 exam to a pass/fail grading system in 2022 has resulted in a shift in emphasis toward USMLE Step 2 Clinical Knowledge (CK) scores [1,4,5]. As residency programs now rely more heavily on this examination score, applicants have been forced to prioritize their performance on USMLE Step 2 CK [5-7]. This change has altered the evaluative landscape, prompting both medical schools and applicants to recalibrate their preparation strategies. In an environment where every point feels like it can be the difference between a successful match and an unsuccessful application, the importance of Step 2 CK cannot be understated. In parallel with the increasing emphasis on examination performance, the research experiences of residency applicants across many specialties have also increased [1,8-10]. Applicants are not only focusing on excelling in clinical examinations but are also investing significant time and effort into scholarly activities. In this retrospective study, we analyze trends in research productivity, defined as number of publications, and Step 2 CK scores for dermatology applicants between 2020 and 2024. This study aims to offer valuable insight for not only applicants but also for advisors and program directors navigating an increasingly competitive and evolving resident selection process.

## Materials and methods

In this retrospective analysis of dermatology residency data from the National Resident Matching Program (NRMP), the Charting Outcomes website feature was used to extract data for the years 2020 to 2024. The data was filtered to only include dermatology applicants. Data was included for all available dermatology residency applicants: MD, DO, US International Medical Graduates (IMG), Non-US IMG, Canadian, and Fifth Pathway applicants. The primary focus was to evaluate trends in USMLE Step 2 CK scores and the number of research publications among matched applicants compared to their unmatched counterparts. Data was extracted directly from the NRMP Charting Outcome website and summarized in Table 1 for both matched and unmatched applicants. The analysis was limited to 434 applicants in 2020, 421 applicants in 2021, 454 applicants in 2022, 497 applicants in 2023, and 446 applicants in 2024 who self-reported their application data to the NRMP. A Chi-square test of independence was conducted at a significance level of 0.05 to determine whether an applicant's match status had a significant relationship with a STEP 2 CK score above 250, and also whether the applicant had over 25 publications.

**Table 1:** Match data (number of applicants matched and unmatched) stratified by Step 2 CK score for 2020-2024 CK: Clinical Knowledge

Year	Number of Publications	Step 2 CK Score							
		Matched vs. Unmatched	<200	200-209	210-219	220-229	230-239	240-249	250 and Above
2024	No publications	Matched	0	0	0	1	0	0	0
		Unmatched	0	0	0	0	0	0	1
	Less than 3	Matched	0	0	1	0	0	0	0
		Unmatched	0	0	0	2	2	2	4
	3-5	Matched	0	0	0	0	4	1	8
		Unmatched	0	0	1	0	1	5	3
	5-10	Matched	0	0	0	1	2	4	19
		Unmatched	0	0	3	4	7	11	17
	11-15	Matched	0	0	1	1	1	6	33
		Unmatched	0	0	2	2	4	8	13
	16-20	Matched	0	0	0	2	3	3	28
		Unmatched	0	0	0	0	0	10	14
	21-25	Matched	0	0	0	0	3	4	29
		Unmatched	0	0	1	0	4	2	12
	More than 25	Matched	0	0	1	1	4	24	91
		Unmatched	0	0	0	5	5	8	17
2023	No publications	Matched	0	0	0	0	0	0	1
		Unmatched	0	0	0	0	0	0	1
	Less than 3	Matched	0	0	0	0	0	0	1
		Unmatched	1	0	1	1	1	2	1
	3-5	Matched	0	0	0	1	2	4	12
		Unmatched	0	1	1	1	6	0	13
	5-10	Matched	0	0	0	1	5	5	34
		Unmatched	0	1	1	2	7	9	18
	11-15	Matched	0	0	1	1	5	8	38
		Unmatched	0	0	0	3	3	10	19
	16-20	Matched	0	0	1	0	5	7	44
		Unmatched	0	0	1	1	4	6	9
	21-25	Matched	0	0	0	0	2	9	28
		Unmatched	0	0	1	0	1	3	11
	More than 25	Matched	0	0	1	2	1	17	84
		Unmatched	0	0	1	3	5	7	21
2022	No publications	Matched	0	0	0	1	0	0	0
		Unmatched	0	0	0	0	1	0	1
	Less than 3	Matched	0	0	0	1	0	1	4
		Unmatched	1	0	0	2	1	4	8
	3-5	Matched	0	0	0	0	2	4	19
		Unmatched	0	0	1	4	2	6	8
	5-10	Matched	0	0	0	2	2	15	31
		Unmatched	0	0	1	4	2	3	14
	11-15	Matched	0	0	0	0	2	12	40
		Unmatched	0	0	0	3	2	7	24
	16-20	Matched	0	0	0	1	1	4	48
		Unmatched	0	0	0	0	7	6	7
	21-25	Matched	0	0	0	1	2	8	25
		Unmatched	0	0	1	1	0	4	2
	More than 25	Matched	0	0	1	0	7	12	55
		Unmatched	0	0	2	1	5	6	12
2021	No publications	Matched	0	0	0	0	0	0	3
		Unmatched	0	0	0	0	0	0	0
	Less than 3	Matched	0	0	0	0	1	3	4
		Unmatched	0	0	0	0	2	5	1
	3-5	Matched	0	0	0	1	1	5	21
		Unmatched	0	1	0	0	2	9	12
	5-10	Matched	0	0	1	1	1	7	38
		Unmatched	0	0	0	4	1	8	14
	11-15	Matched	0	0	0	0	7	13	52
		Unmatched	0	0	0	1	3	5	8
	16-20	Matched	0	0	0	1	5	12	20
		Unmatched	0	0	0	1	1	4	4
	21-25	Matched	0	0	0	2	1	3	28
		Unmatched	0	0	0	2	2	1	4
	More than 25	Matched	0	0	0	2	7	7	64
		Unmatched	0	0	2	0	6	4	3
2020	No publications	Matched	0	0	0	0	0	0	2
		Unmatched	0	0	1	0	1	0	1
	Less than 3	Matched	0	0	0	0	0	2	11
		Unmatched	0	0	0	3	2	1	4
	3-5	Matched	0	0	0	0	1	6	15
		Unmatched	0	0	0	2	1	6	8
	5-10	Matched	0	0	0	1	3	21	50
		Unmatched	0	0	0	2	4	3	7
	11-15	Matched	1	0	0	0	3	8	43
		Unmatched	0	0	0	1	4	2	9
	16-20	Matched	0	0	0	1	4	13	35
		Unmatched	0	0	0	1	3	1	2
	21-25	Matched	0	0	1	1	2	9	37
		Unmatched	1	1	0	0	1	1	1
	More than 25	Matched	0	0	1	4	5	13	57
		Unmatched	0	0	1	1	0	3	5

## Results

**Figure 1:**
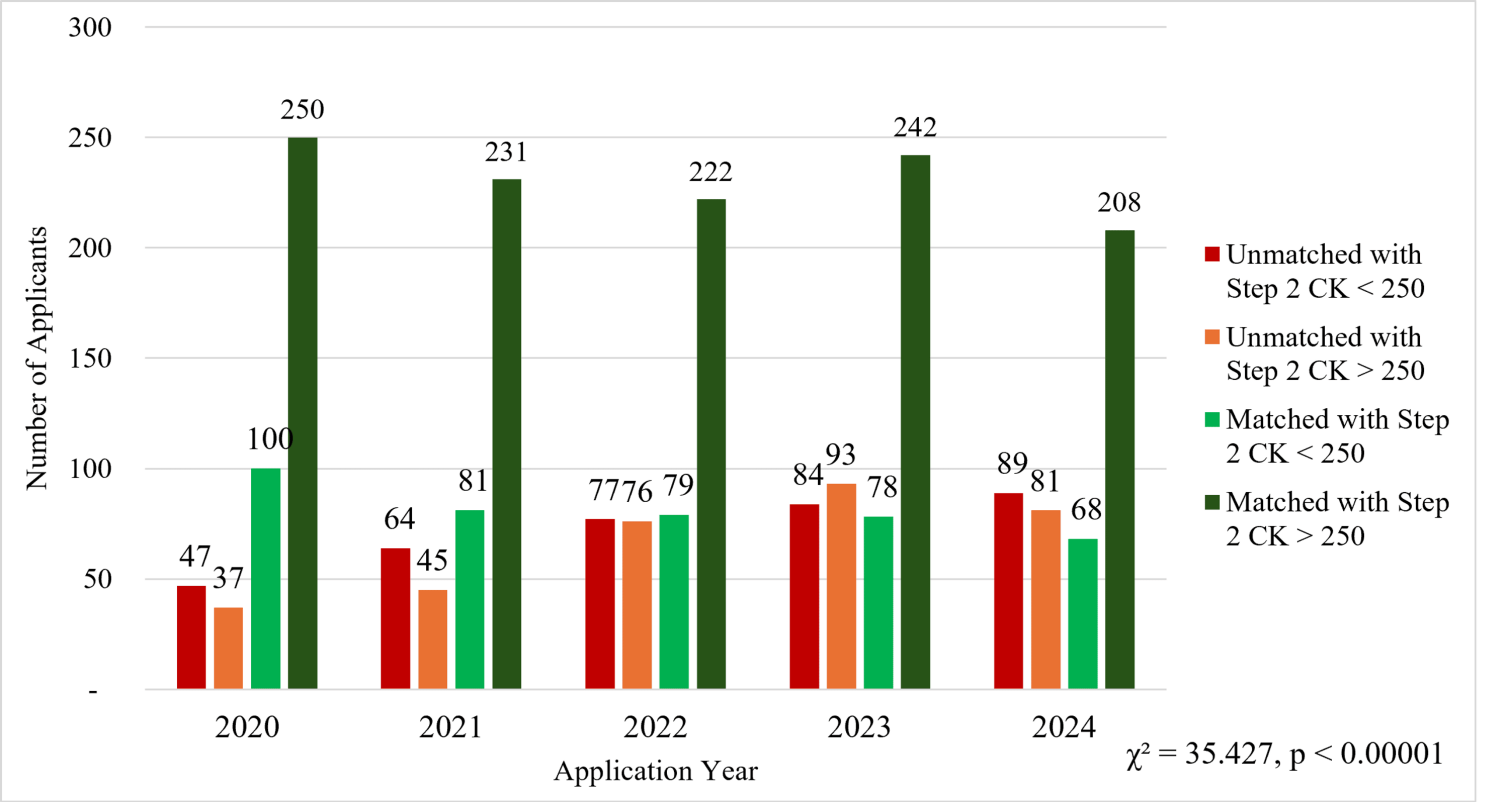
Characteristics of matched and unmatched dermatology residency applicants based on Step 2 CK score, 2020-2024 CK: Clinical Knowledge

From 2020 to 2024, the trends in dermatology match outcomes have shown variability in the relationship between Step 2 CK scores and match success (Figure 1). The number of applicants with a Step 2 CK score greater than 250 and a successful match declined from 250 in 2020 to 208 in 2024. Conversely, the number of unmatched applicants with a Step 2 CK score greater than 250 has risen from 37 in 2020 to 81 in 2024. Similarly, the number of applicants with Step 2 CK scores below 250 who went unmatched increased from 47 in 2020 to 89 in 2024. The number of matched applicants in this category declined from 100 to 68 over the same time. A chi-square test determined that there was a significant relationship between an applicant's match status and whether they had a STEP 2 CK score above 250 (χ² = 35.427, p < 0.00001). These trends suggest that while an above-average Step 2 CK score remains an important component of a successful application, its predictive value for a successful match outcome has diminished over this period.

**Figure 2:**
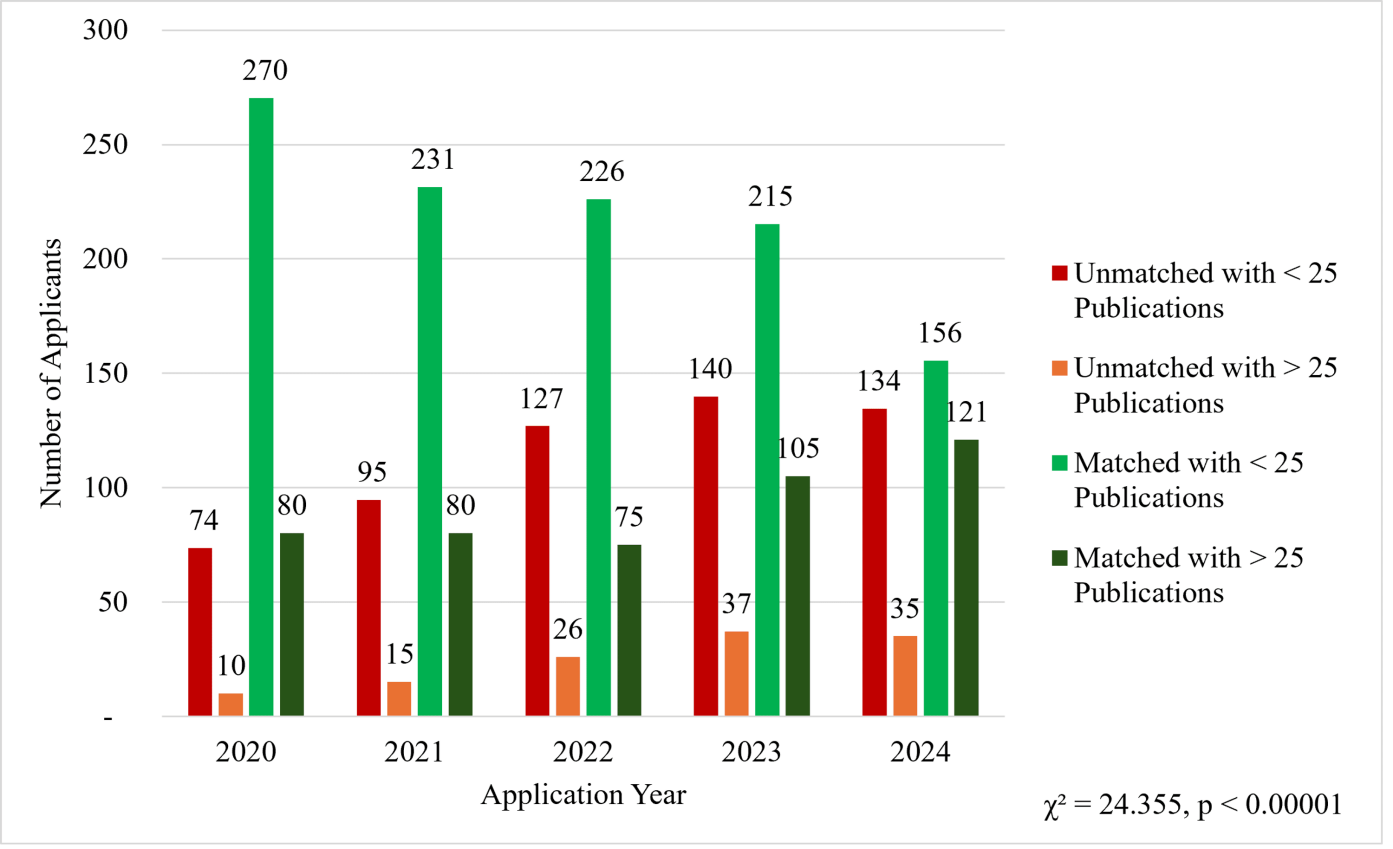
Characteristics of matched and unmatched dermatology residency applicants based on research publications, 2020-2024

Figure 2 demonstrates the number of applicants from 2020 to 2024 stratified by match status and the number of publications on their application. Over this period, there is a notable increase in the number of applicants with fewer than 25 publications who went unmatched, increasing from 74 in 2020 to 134 in 2024. Unmatched applicants with more than 25 publications also increased from 10 in 2020 to 35 in 2024. On the other hand, successfully matched applicants with fewer than 25 publications have decreased from 270 in 2020 to only 156 in 2024. Also, the number of matched applicants with greater than 25 publications has increased from 80 in 2020 to 121 in 2024. A chi-square test also determined that there was a significant relationship between an applicant's match status and whether they had over 25 publications (χ² = 24.355, p < 0.00001). This trend suggests that the higher number of publications is increasingly correlated with a successful outcome.

**Figure 3:**
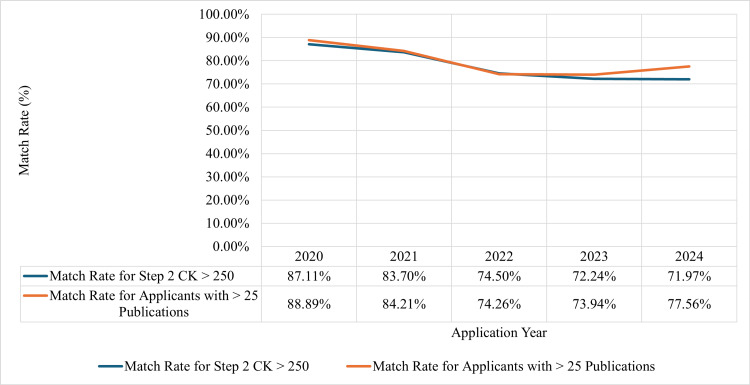
Match rate for high-performing dermatology applicants, 2020-2024 CK: Clinical Knowledge

The overall match rate of applicants based on Step 2 CK performance and the match rate among applicants with extensive publications have decreased since 2020 (Figure 3). In the 2020 match cycle, the match rate for dermatology applicants with a Step 2 CK score greater than 250 was 87%; however, this match rate decreased to 72% in the 2024 application cycle. In addition, the match rate for applicants with over 25 publications decreased from 89% in 2020 to 77.3% in 2024.

## Discussion

The analysis of dermatology residency match data from 2020 to 2024 reveals trends that tell the story of the evolving landscape of the application process. The observed increase in Step 2 CK scores among matched applicants is indicative of the shifting focus within residency selection, particularly following the transition of the Step 1 exam to a pass/fail system [1,4]. As a result, both applicants and residency programs have had to adjust their strategies, leading to an intensified focus on clinical knowledge and test preparation. This change has elevated the importance of Step 2 CK scores and heightened the pressure on applicants to perform exceptionally well on a single examination [5-7]. The shift to a pass/fail grading system for Step 1 has moved the emphasis to other measures such as Step 2 CK scores and research output [11]. This move has presented hurdles for applicants, particularly those from less traditional backgrounds, such as osteopathic or IMGs, who historically relied on strong Step 1 results to distinguish themselves [7,11,12]. Additionally, program directors have expressed worries about the difficulties of distinguishing candidates without quantitative Step 1 scores [11,12].

Our findings are consistent with previous studies that have noted a compensatory rise in the weight of Step 2 CK scores [5-7]. In the past, applicants had two separate scores to distinguish their abilities. Now, low performance on a single testing day may lead to an unsuccessful match. The observed trends among matched and unmatched candidates may indicate that, while a high Step 2 CK score is frequently seen as a positive indicator, it does not always correlate with a successful match. If anything, a low step score may harm rather than aid a person's application.

In addition to the increased emphasis on Step 2 CK scores, the trend towards a higher number of research publications among matched applicants underscores a broader shift toward valuing scholarly productivity in dermatology [13]. The data indicates that more matched candidates committed time to research activities [13,14]. This trend is made more apparent with the growing pressure to pursue dedicated research years to further bolster an applicant’s research experiences [1,15]. This trend aligns with the growing recognition within academic medicine that research not only enhances critical thinking and problem-solving skills but also contributes to the advancement of the specialty as a whole [1]. The observed pattern of an increased number of research experiences among matched and unmatched applicants suggests that high research output appears to bolster match potential. However, it is also not a sole factor as some high-achieving candidates remain unmatched.

The overall match rate decline of applicants with a Step 2 CK greater than 250 or with extensive publications could suggest changes in applicant volume, competitiveness, evolving selection criteria, or broader trends in the field that have altered the weight of traditional academic metrics. The growing emphasis on research output parallels a broader trend in academic medicine, where scholarly work is becoming more valued [13-15]. However, this may disadvantage students who attend colleges with fewer research options or who are unable to devote time to research owing to financial or personal restrictions.

A large limitation of our study is that the NRMP data is self-reported by applicants without mechanisms in place to verify the responses provided [16]. NRMP data is also not controlled for confounding variables, like letters of recommendation, grades, and success on an interview day. However, even with these limitations, these trends raise several important questions and concerns given the increased focus on research and examination scores. One concern is the increased stress and workload on applicants who are striving to excel in both high-pressure examinations and demanding research activities. The expectation to maintain a high Step 2 CK score while simultaneously producing numerous publications could lead to burnout and impact overall medical student well-being [6,7]. This trend might inadvertently disadvantage applicants from institutions with limited research opportunities or those without access to quality mentors in the field. Additionally, virtual interviews were implemented during the COVID-19 pandemic and are likely to have influenced application trends and outcomes [17,18]. They may have decreased geographic bias and financial pressure, but they also restricted applicants' capacity to build personal contacts with teachers and evaluate program culture [18,19].

Despite these challenges, the trend toward increased academic and research achievements among dermatology applicants may also be viewed as a positive change. A strong foundation in both clinical knowledge and research skills is necessary for the advancement of dermatological knowledge and patient care. As such, the heightened focus on these metrics could promote continuous learning, innovation, and evidence-based practice in dermatology [1,20]. However, while Step 2 scores and research experiences remain important for residency selection, many other factors contribute to a successful match, including application year, signaling, number of programs applied to, letters of recommendation, connections, and interview experience. Future studies should examine how non-academic metrics impact an applicant’s match status. Future studies can also evaluate whether similar trends have been observed in other specialties following the transition of Step 1 to pass/fail.

## Conclusions

From 2020 to 2024, dermatology residency applicants have seen rising Step 2 CK scores and research output, underscoring the competitiveness of the specialty. While Step 2 CK scores and research experiences are important parts of the application to dermatology residency, they are not the only factors used by programs to choose applicants. Future applicants must strategically focus on excelling in Step 2 CK and securing meaningful research experiences while also bolstering their applications in other ways to maximize their chances of a successful match.

## References

[1] Elliott B, Carmody JB (2023). Publish or perish: the research arms race in residency selection. J Grad Med Educ.

[2] Korman AM, Grant-Kels JM (2018). Applying to dermatology residency: an ethical approach to an inherently unethical process. Int J Womens Dermatol.

[3] Whiteside M, Bolen R, Szymanski T, Farsi M, Nem S, Carboni A, Marroquin N (2025). Navigating the transition: the impact of step/level 1 pass/fail grading on dermatology residency match outcomes. J of Skin.

[4] (2025). USMLE Step 1 transition to pass/fail only score reporting. https://www.usmle.org/usmle-step-1-transition-passfail-only-score-reporting.

[5] Lubell J (2025). After Step 1 scoring change, what residency programs look for now. AMA. May 21.

[6] Khalil S, Jose J, Welter M (2023). The importance of USMLE step 2 on the screening and selection of applicants for general surgery residency positions. Heliyon.

[7] Rothka AJ, Nguyen M, King TS, Choi KY (2024). Impact of USMLE pass/fail step 1 scoring on current medical students. J Med Educ Curric Dev.

[8] Samadzadeh Tabrizi N, Shen M, Shapeton AD, Doshi I, Liu J, Fabian T, Chan P (2025). Research productivity among applicants who matched into an integrated thoracic surgery residency program: a bibliographic review. JTCVS Open.

[9] Varieur BM, White RC, Bono CM (2025). Increasing research productivity and Step 2 score among matched orthopaedic surgery residents: a forecasting analysis to 2040. J Am Acad Orthop Surg.

[10] Zhou B, Srinivasan N, Nadkarni S, Taruvai V, Song A, Khouri AS (2022). Current trends of research productivity among students matching at top ophthalmology programs. J Acad Ophthalmol (2017).

[11] Choi P, Langenau E, Roberts M, Blalock TW (2023). Perspectives of dermatology program directors on the impact of Step 1 pass/fail. Cureus.

[12] Mun F, Scott AR, Cui D, Chisty A, Hennrikus WL, Hennrikus EF (2021). Internal medicine residency program director perceptions of USMLE Step 1 pass/fail scoring: a cross-sectional survey. Medicine (Baltimore).

[13] Manstein SM, Laikhter E, Kazei DD, Comer CD, Shiah E, Lin SJ (2023). The upcoming pass/fail USMLE Step 1 score reporting: an impact assessment from medical school deans. Plast Surg (Oakv).

[14] Salehi PP, Azizzadeh B, Lee YH (2021). Pass/Fail scoring of USMLE Step 1 and the need for residency selection reform. Otolaryngol Head Neck Surg.

[15] Lenze NR, Benjamin WJ, Kay HG (2024). Association of USMLE Step 1 pass/fail reporting with interview and match outcomes. J Surg Educ.

[16] (2025). Charting Outcomes™: USMLE Step 2 CK exam baseline. https://www.nrmp.org/match-data/2024/08/charting-outcomes-usmle-step-2-ck-exam-baseline/#:~:text=Inclusive-,(All),USMLE%20Step%202%20CK%20Score.

[17] Domingo A, Rdesinski RE, Stenson A (2022). Virtual residency interviews: applicant perceptions regarding virtual interview effectiveness, advantages, and barriers. J Grad Med Educ.

[18] Beesley H, Pernar L, Kettoola Y, Hess D (2023). The association between virtual interviewing and geographical distribution of matched residency programs for general surgery applicants. J Surg Educ.

[19] Rinderknecht FB, Bailey KC, Novack DE (2024). Changing perceptions about the dermatology residency application process among applicants and program directors: 2020-2022. Cureus.

[20] Anderson JM, Wenger D, Johnson AL (2021). Publication trends and their relationship with academic success among dermatology residents: cross-sectional analysis. JMIR Dermatol.

